# Bacterial Community Affects Toxin Production by *Gymnodinium catenatum*

**DOI:** 10.1371/journal.pone.0104623

**Published:** 2014-08-12

**Authors:** Maria E. Albinsson, Andrew P. Negri, Susan I. Blackburn, Christopher J. S. Bolch

**Affiliations:** 1 National Centre for Marine Conservation and Resource Sustainability, Australian Maritime College, University of Tasmania, Launceston, Tasmania, Australia; 2 Commonwealth Scientific and Industrial Research Organisation, Marine and Atmospheric Research, Hobart, Tasmania, Australia; 3 Australian Institute of Marine Science, Townsville, Queensland, Australia; University of New South Wales, Australia

## Abstract

The paralytic shellfish toxin (PST)-producing dinoflagellate *Gymnodinium catenatum* grows in association with a complex marine bacterial community that is both essential for growth and can alter culture growth dynamics. Using a bacterial community replacement approach, we examined the intracellular PST content, production rate, and profile of *G. catenatum* cultures grown with bacterial communities of differing complexity and composition. Clonal offspring were established from surface-sterilized resting cysts (produced by sexual crosses of strain GCDE06 and strain GCLV01) and grown with: 1) complex bacterial communities derived from each of the two parent cultures; 2) simplified bacterial communities composed of the *G. catenatum*-associated bacteria *Marinobacter* sp. strain DG879 or *Alcanivorax* sp. strain DG881; 3) a complex bacterial community associated with an untreated, unsterilized sexual cross of the parents. Toxin content (STX-equivalent per cell) of clonal offspring (134–197 fmol STX cell^−1^) was similar to the parent cultures (169–206 fmol STX cell^−1^), however cultures grown with single bacterial types contained less toxin (134–146 fmol STX cell^−1^) than offspring or parent cultures grown with more complex mixed bacterial communities (152–176 fmol STX cell^−1^). Specific toxin production rate (fmol STX day^−1^) was strongly correlated with culture growth rate. Net toxin production rate (fmol STX cell^−1^ day^−1^) did not differ among treatments, however, mean net toxin production rate of offspring was 8-fold lower than the parent cultures, suggesting that completion of the sexual lifecycle in laboratory cultures leads to reduced toxin production. The PST profiles of offspring cultures were most similar to parent GCDE06 with the exception of cultures grown with *Marinobacter* sp. DG879 which produced higher proportions of dcGTX2+3 and GC1+2, and lower proportions of C1+2 and C3+4. Our data demonstrate that the bacterial community can alter intracellular STX production of dinoflagellates. In *G. catenatum* the mechanism appears likely to be due to bacterial effects on dinoflagellate physiology rather than bacterial biotransformation of PST toxins.

## Introduction

Paralytic shellfish toxins (PST) are neurotoxic alkaloids produced by several dinoflagellates, including a number of species of the genus *Alexandrium*, *Pyrodinium bahamense*, and the unarmoured species *Gymnodinium catenatum* [1], [2], [3], [4], [5]. The toxins consist of the parent compound saxitoxin (STX) and at least 22 derivatives of varying oral toxicity [6].

Putative biosynthetic genes and pathways for PST production are now known from several cyanobacteria [7], [8], [9], and homologous genes have been detected from PST-producing dinoflagellates including *G. catenatum* [10]. There is currently no convincing structural or molecular evidence that heterotrophic bacteria produce PST autonomously yet the bacterial community is known to indirectly influence dinoflagellate PST toxicity [11], [12], [13] either by biotransformation from one PST derivative to another [14], [15], [16] or potentially via their effect on dinoflagellate growth and physiology [17], [18]. For example, axenic *Alexandrium* and *Protogonyaulax* cultures have been shown to contain a higher PST content than their non-axenic counterparts, suggesting that the associated bacterial community may reduce dinoflagellate toxin production under some circumstances [18], [19].

*Gymnodinium catenatum* is capable of producing at least 20 PSTs: the N-sulfocarbamoyl gonyautoxins (GTXs); the N-sulfocarbamoyl-11-hydroxysulfate C-toxins; the hydroxyl-benzoate toxins (GC-toxins); and a number of non-sulfated saxitoxin analogues [5], [20], [21], [22]. The range of PSTs produced varies considerably within and between populations, and culture-induced variation is also evident [13], [21]. The reasons for this high level of variation are poorly understood, but may be due to gene variation in the saxitoxin biosynthesis pathway [23], other genetic factors controlling toxin biosynthesis, or the direct or indirect effects of the microbial community [11], [21], [24].

Laboratory-grown *G. catenatum* cultures are associated with a suite of associated bacteria [11], [24], in which the two -proteobacteria, *Marinobacter* sp. and *Alcanivorax* sp., are constant components [11], [24]. Using strains originally isolated from the Tasmanian *G. catenatum* strain GCDE08 [11], Bolch et al., [25] found that *G. catenatum* has an obligate requirement for bacterial associates and that both *Marinobacter* sp. DG879 and *Alcanivorax* sp. DG881 are capable of supporting survival and growth of the vegetative *G. catenatum* cells. Given this high reliance on bacterial associates, we anticipated that the bacterial community could alter toxin content and/or production of the dinoflagellate cell.

Our earlier studies have found no consistent link between community membership and cellular toxicity [11]; however, these studies did not examine PST production rates and were potentially confounded by isolation, geographic and culture-related effects. Here we use controlled uni-bacterial community replacement [25] to show that changes to the associated bacterial community modify both PST content and production by the dinoflagellate *G. catenatum*.

## Materials and Methods

### Production of resting cysts

Two sexually compatible, PST-producing, Tasmanian strains, GCDE06 (CS-301/06) and GCLV01 (CS-800), were provided by the Australian National Algae Culture Collection (ANACC; http://www.csiro.au/ANACC). Sexual crosses were carried out in sterile 55 mm diameter plastic Petri dishes containing 10 mL of nitrate and phosphate deficient GSe medium [26] and incubated at 21°C±2°C, at 80 µmol PAR m^−2 ^sec^−1^ with a 18∶6 h light:dark cycle for 3 weeks to promote cyst formation. Resting cysts were harvested by manual micropipetting, washed several times in fresh GSe medium, surface-sterilized in 0.5% H_2_O_2,_ and washed 3 times to remove residual H_2_O_2_ [25]. Successful surface-sterilization of cysts was checked by spread-plating 10 µL of the sterilized sample onto ZM1 agar incubated at 24°C for 4 days. If bacterial growth was observed after incubation, the cysts were discarded.

### Unibacterial and community replacement cultures

A community replacement approach [25] was used to establish a series of clonal offspring cultures with the following modified bacterial communities: 1) complex bacterial communities derived from each of the two parent cultures GCDE06 and GCLV01; 2) simplified uni-bacterial communities of the *G. catenatum*-associated bacteria *Marinobacter* sp. DG879 or *Alcanivorax* sp. DG881; or 3) a complex bacterial community derived from unsterilized cysts from a sexual cross of both parent cultures.

Table 1 summarises all cultures established in this study. Bacterial strains *Marinobacter* sp. DG879 and *Alcanivorax* sp. DG881 were originally isolated from the *G. catenatum* strain GCDE08 [24] and maintained on modified ZoBell’s Marine agar (ZM1) [25], [27] at 20°C in total darkness. Groups of ten to fifteen sterilized *G. catenatum* cysts were each placed into 55 mm diameter sterile polystyrene Petri dishes containing 10 mL GSe medium using a micropipette. A specific bacterial community was then added to each of the Petri dishes, with triplicate Petri dishes established for each of the four different bacterial communities as follows: Community 1) 1 mL of 10^5^ cells mL^−1^ of *Marinobacter* sp. DG879; Community 2) 1 mL of 10^5^ cells mL^−1^ of *Alcanivorax* sp. DG881; Community 3) 1 mL of 10^5^ cells mL^−1^ of a 5 µm filtrate from the non-axenic parent strain GCDE06; and Community 4) 1 mL of 10^5^ cells mL^−1^ of a 5 µm filtrate from the non-axenic parent strain GCLV01. Two sets of experimental control cultures were also established as follows:

**Table 1:** Summary of established *G. catenatum* cultures used in this study.

CultureTreatmentname	Original*G. catenatum*cultures	Bacterialcommunity inculture	Bacterialconcentrationat time of cultureestablishment
	**Parent cultures**		
DE06	GCDE06	Mixed culture-associatedcommunity	∼10^5^ cells mL^−1^
LV01	GCLV01	Mixed culture-associatedcommunity	∼10^5^ cells mL^−1^
	**Cyst-derived cultures**		
Gc/Mar*	GCDE06×GCLV01	*Marinobacter* sp. DG879	10^5^ cells mL^−1^
Gc/Alc*	GCDE06×GCLV01	*Alcanivorax* sp. DG881	10^5^ cells mL^−1^
DE06 filtrate	GCDE06×GCLV01	Filtrate (5 µm) from thenon-axenic parent strainGCDE06	10^5^ cells mL^−1^
LV01 filtrate	GCDE06×GCLV01	Filtrate (5 µm) fromthe non-axenic parentstrain GCLV01	10^5^ cells mL^−1^
Positive control	GCDE06×GCLV01	Mixed culture-associatedcommunity fromboth parent cultures	∼10^5^ cells mL^−1^
Sterilitycontrol**	GCDE06×GCLV01	None- sterilizedcystsplaced in sterileseawater	N/A

The seven final cultures included two parent cultures and five cyst-derived cultures with altered bacterial communities.

*Gc in the culture/treatment name is short for *Gymnodinium catenatum.*

**The sterility control is non-viable as *G.catenatum* cannot grow axenically. Used as a control to assess contamination by bacteria from intracellular or other sources.

1. Positive controls: Offspring cultures established from unsterilized cysts allowed to excyst in sterile GSe medium. These controls are equivalent of offspring from a typical crossing experiment. Survival and growth indicates the resting cyst viability.
2. Sterility controls: Surface-sterilized cysts germinated in sterile (0.2 µm) filtered seawater. Survival after germination is not expected as *G. catenatum* cannot grow in the absence of bacteria [25]. Survival and growth indicates bacterial contamination either from failed surface-sterilization, intracellular bacteria released at germination, or from other sources such as contaminated growth medium.

Petri dishes were incubated for 3 weeks at 21°C±2°C, 80 µmol PAR m^−2 ^sec^−1^ with a 18∶6 h light:dark cycle to allow excystment of the resting cysts. Clonal isolates were established from the mixed offspring cultures by dilution in sterile GSe medium (to 1 cell mL^−1^) and aseptic distribution of 1 mL aliquots into wells of sterile 24-multiwell plates containing 2 mL sterile GSe medium. Wells were examined using a Leitz Labovert FS inverted microscope (200× magnification), those containing a single cell/chain were labelled for later isolation, and the plate incubated for a further week. Clonal cultures were established from labelled wells by aseptic transfer into 50 mL Erlenmeyer flasks containing 25 mL GSe medium, and 2 weeks later to 50 mL flasks containing 40 mL GSe medium.

A total of 5 clonal isolates were established for each of the 5 bacterial treatments, together with 5 clonal cultures of each of the two parent strains (GCDE06 and GCLV01). The clonal cultures were maintained on three week aseptic transfer intervals in 50 mL Erlenmeyer flasks at 18°C, 80 µmol PAR m^−2 ^sec^−1^ with an 18∶6 h light:dark cycle (Phillips cool-white fluorescent). Transfers to fresh medium were made every 2 weeks.

### Toxin production of cultures

Cultures for toxin content and production rate estimates were transferred to 175 mL Erlenmeyer flasks and maintained for 50 days under the same light and temperature conditions described earlier to establish the period of exponential growth phase of cultures prior to the toxin production experiment. At Day 0, 10 mL of late exponential phase cultures was inoculated into 150 mL of sterile GSe medium and the remaining inoculum culture volumes retained for triplicate cell counts (Leitz Labovert FS microscope, 200× magnifications) and toxin analysis. Subsequent cultures were grown at 18°C and 80 µmol PAR m^−2 ^sec^−1^ light with a 18∶6 h light:dark cycle until Day 25 (late-exponential phase) and harvested for toxin analysis. Cell concentration was estimated every 5 days from triplicate cell counts using a Sedgwick-Rafter counting chamber (Graticules Ltd, UK). Culture samples (100 mL) were filtered through precombusted (400°C, 4 h) 47 mm GF/C (Whatman) filters, placed in 15 mL screw-cap polycarbonate centrifuge tubes containing 5 mL of 0.05 M acetic acid. Samples were sonicated for 30 s on ice several times using an ultrasonic cell disruptor (Braunsonic, 150 W, 5 mm probe), centrifuged at 5000×g for 5 min, the supernatants filtered through 0.45 µm filters, and then frozen at −20°C until analysis.

### HPLC analysis of PSTs

PSTs including C-toxins, gonyautoxins (GTX) and the hydroxybenzoate (GC) toxins were analysed by HPLC using the methods of Negri and Jones [28] and Negri et al. [20]. Briefly, toxins were separated using a Waters 600 HPLC, with post-column reactor (Pickering PCX 5100) using a 5 µm, 250 mm×4.6 mm Alltima ODS column (Alltech, IL, USA) with a flow rate of 0.8 mL min^−1^. Post-column oxidation was performed according to the method of Oshima et al. [4]. Derivative PST fluorescence was detected with a Linear LC305 spectrofluorometric detector (excitation at 330 nm and emission at 390 nm). The retention times and fluorescent intensity of the PSTs were compared with PST standards (NRC, Canada) and identity of PST compounds confirmed by sample spiking experiments and removing post-column oxidation and observing the disappearance of peaks. The quantification of toxins was achieved by comparing peak areas with those of authentic standards and combining this data with cell counts from the time of harvest then converting to total PST expressed as fmol cell^−1^. As the proportion of α-epimers is often very small and some interconversion is expected, concentrations of epimer pairs were combined comparing toxin profiles.

### Calculations and statistical analyses

Specific growth rates (µ) of the cultures from Day 0 to Day 25 were calculated over the exponential growth phase (Day 0 to Day 25) using the equation:
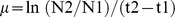
where *N1* and *N2* = cell concentration at time 1 (*t1*) and time 2 (*t2*) respectively [29]. Specific toxin production rates (*µ_tox_*) and net toxin production rates between Day 0 and 25 were calculated using the equations and methods described by Anderson et al. [30]. Briefly, the STX content of cells (fmol cell^−1^) at Day 0 and at Day 25 was multiplied by *N_t_* (cell concentration at time *t*) to yield *T_t_*, the total toxin concentration (fmol STX mL^−1^ culture) at time *t.* Toxin concentrations (fmol STX mL^−1^) at Day 0 were calculated from the STX content (fmol cell^−1^) of the inocula and included a correction for dilution at inoculation. Values of *T* were then used to calculate specific toxin rate *µ_tox_* over each time interval:



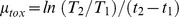



The net toxin production rate *R_tox_* (fmol toxin cell^−1 ^d^−1^) was determined using:
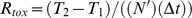
where *N'* is the *ln* average of the cell concentration (below):



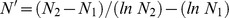
and *Δt* ( = *t_2_*−*t_1_*) the interval between Day 0 and Day 25. The *ln* average concentrations are used to account for exponential growth over the time period *t_1_*−*t_2_* [30].

The correlation between the specific toxin production rate and algal growth rate was done using linear regression. Significant differences in toxin production among bacterial culture treatments were compared using one-way ANOVA using the statistical software package R (Version 2.9.0), with treatment and time (Day 0 and 25) as factors, followed by Tukey’s post hoc tests of significance.

Differences in culture toxin profiles were examined using PERMANOVA+ [31], a multivariate analysis of variance with significance testing by random permutation, as implemented in the PRIMER-6 software package (www.primer-e.com). The toxin profile dataset (mol% STX, Day 25) was standardised prior to analysis to account for unequal variances among treatments. Canonical analysis of principal coordinates (CAP) [32] was performed using principal coordinates (PCO) calculated from a Euclidean distance matrix, and the first two canonical axes plotted. Over-parameterisation of the CAP was limited by restricting the number of PCO axes to that which maximised the leave-one-out allocation to groups [33]. All PERMANOVA and CAP tests used 9999 unrestricted random permutations of the raw data.

## Results

### Culture growth dynamics

Both the positive and the sterility control cultures behaved as expected. The sterility control did not survive, indicating that growth media was not contaminated. The positive controls germinated (in the presence of *G. catenatum* representative bacterial community), survived and were used to establish five replicate cultures. The experimental cultures of *G. catenatum* exhibited average exponential growth rates (K’) between Day 0 and 25 of 0.095±0.005 to 0.14±0.008 Div. day^−1^ with no significant variation among cultures grown with different bacterial communities (Table 2) (F = 1.96, df = 6, p = 0.092).

**Table 2:** Specific growth rate (µ) of *Gymnodinium catenatum* (Day 0 to Day 25) when grown with different microbial communities.

Culture	Specific growth rate (µ; ±SE)
DE06	0.114 (±0.005)
LV01	0.073 (±0.006)
Gc/Mar	0.097 (±0.004)
Gc/Alc	0.127 (±0.008)
Gc/DE06 filtrate	0.084 (±0.004)
Gc/LV01 filtrate	0.104 (±0.005)
Positive control	0.077 (±0.006)

### Toxin content and production

**Figure 1:**
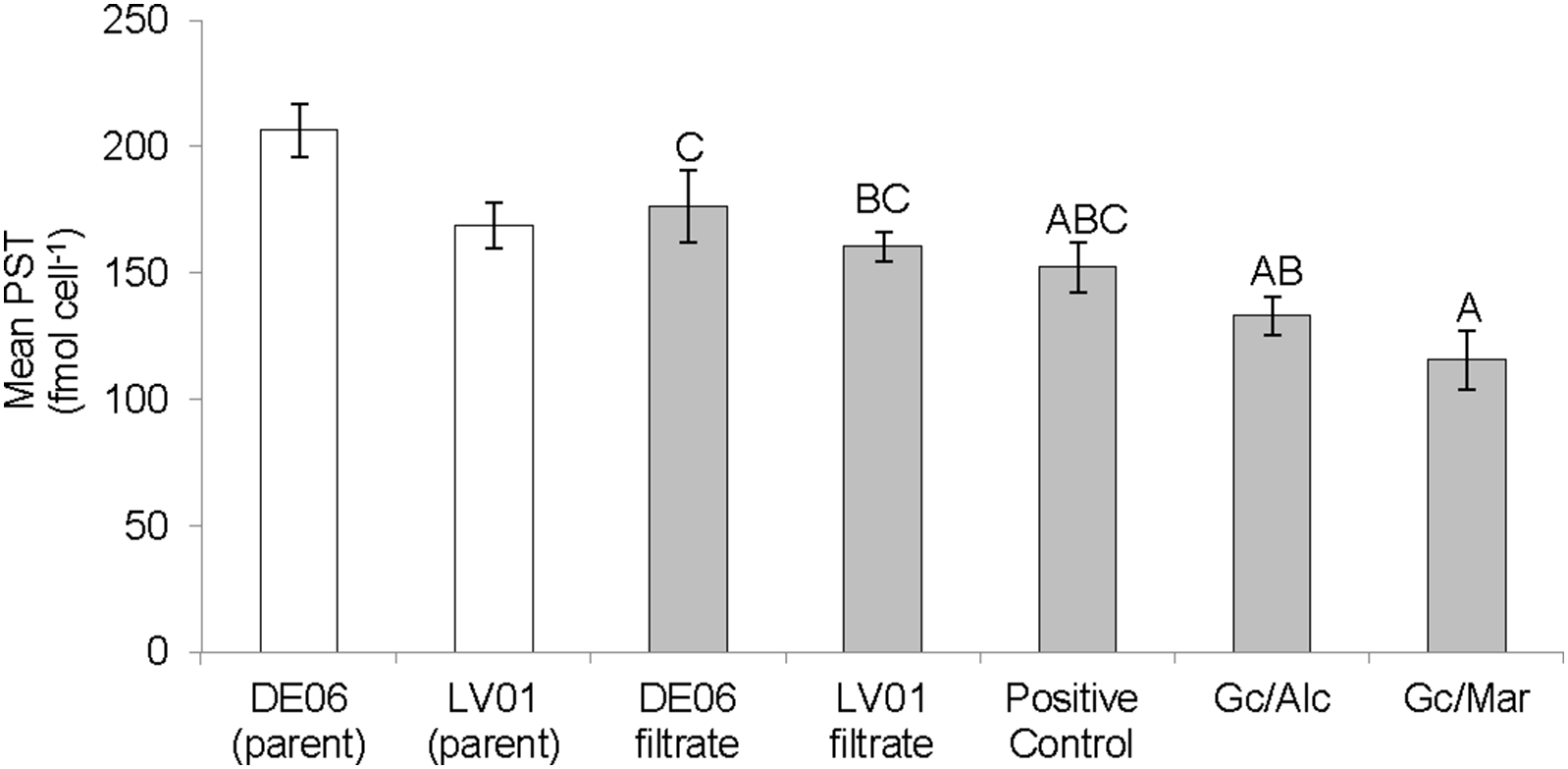
Toxin content (fmol cell^−1^) between Day 0 and Day 25 of *G. catenatum* cultures grown with different bacterial communities. Day 0 and Day 25 toxin content data for the offspring cultures were pooled as there was no significant difference. Toxicity of parent cultures (Day 25) included for comparison only. Superscripts indicate significant differences (p<0.05), means labelled with the same letter are not significantly different. Error bars ± SE, n = 5.

**Figure 2:**
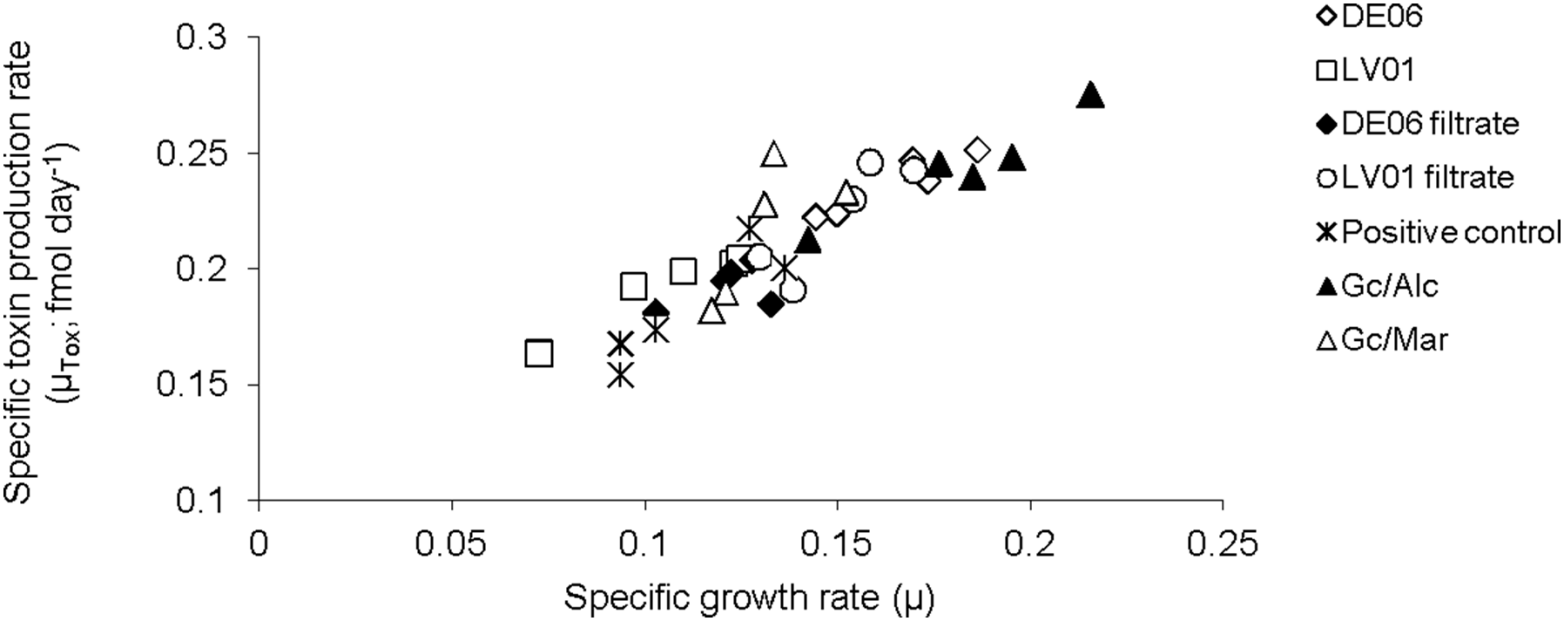
Relationship between specific growth rate (µ) and specific toxin production rate (µ_tox_, fmol day^−1^). The relationship was established using 35 cultures of *Gymnodinium catenatum* measured between Day 0 and Day 25.

**Figure 3:**
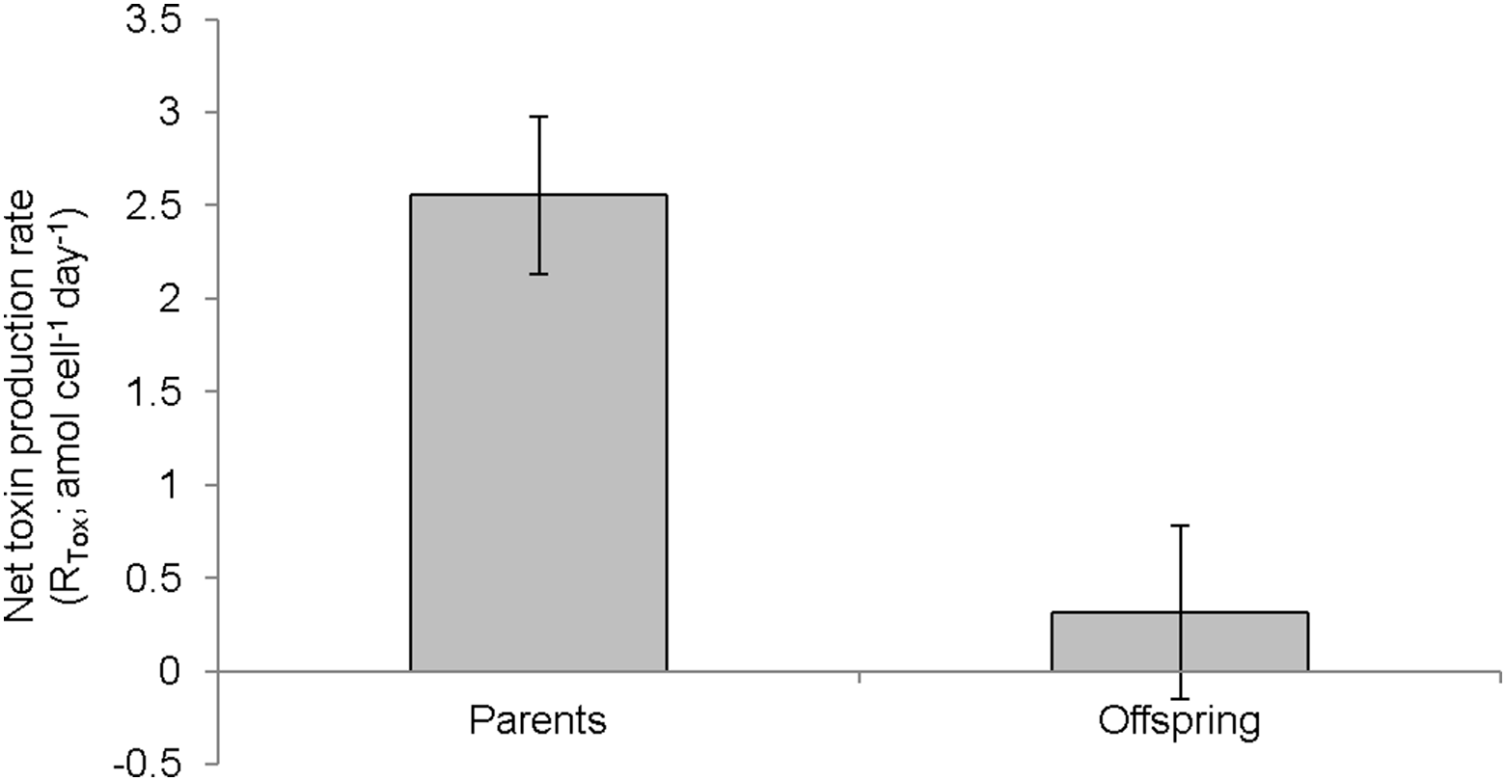
Comparison of the net toxin production rate (R_tox_) of parents (2 strains) and offspring (25 strains). Error bars = ± SE, n = 10 in parents and 25 in offspring.

There was no significant difference in the toxin content of the offspring cultures between Day 0 and 25, (F = 3.416, df = 4, p = 0.072) therefore the data from both time periods was pooled. Cultures established with mixed bacterial communities contained a significantly higher toxin content than cultures grown with single bacterial types (F = 5.89, df = 6, p = 0.007) (Fig. 1). The specific toxin production rate (µ_tox_; fmol day^−1^) varied from 0.07 to 0.13 fmol day^−1^ (F = 6.35, df = 34, p = 0.0003) (Fig. 1) and was strongly correlated with the exponential growth rate of the cultures (df = 34, p = <0.001, R^2^ = 0.82) (Fig. 2). Net toxin production rate (R_tox_; amol cell^−1^ day^−1^) varied considerably among replicates and consequently no significant difference was detected among the different treatments (F = 2.44, df = 6, p = 0.064). However, the net toxin production rate of offspring cultures was almost 8-fold lower than the parent cultures (t = 2.83, df = 33, p = 0.008) (Fig. 3).

### Toxin profiles

**Figure 4:**
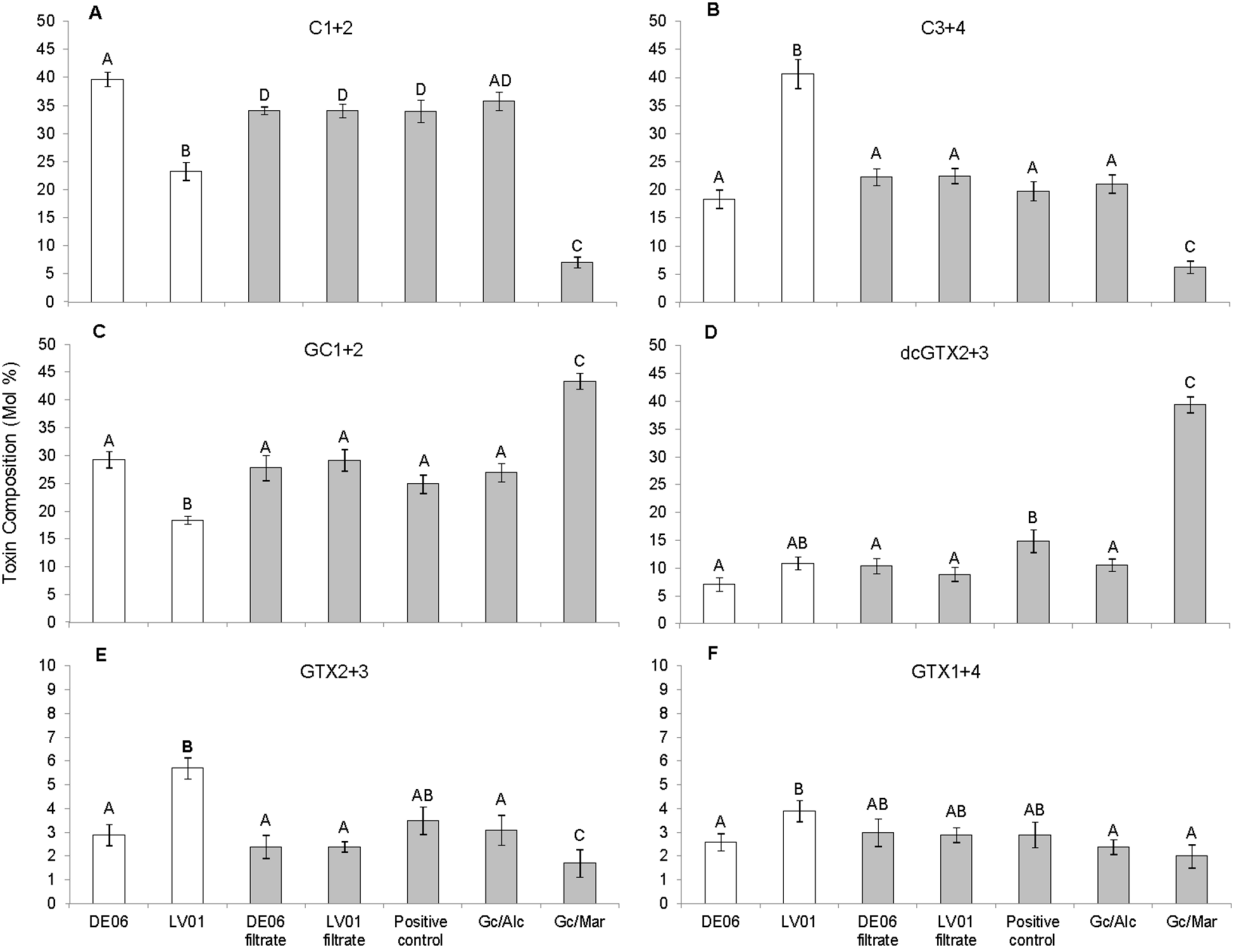
Toxin profiles (mol%) of each toxin group identified. The profiles of the identified toxin groups (C1+2, C3+4, GC1+2, dcGTX2+3, GTX2+3, and GTX1+4) from HPLC analysis on cultures at Day 25 (harvest), Error bars ± SE, n = 5. Superscripts indicate significant differences (p<0.05), means labelled with the same letter are not significantly different.

**Figure 5:**
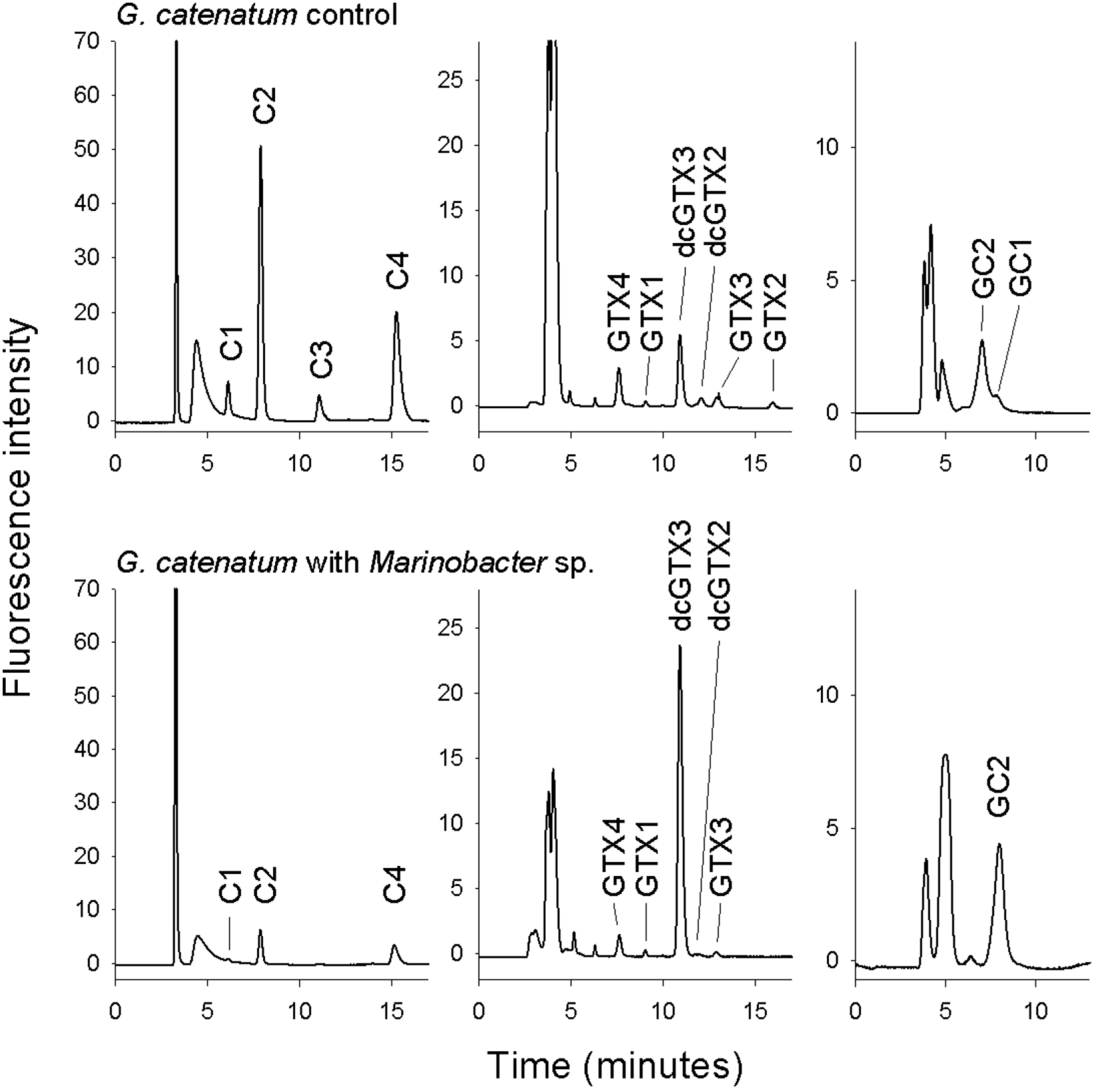
HPLC chromatogram comparison. Comparison of (a) *Gymnodinium catenatum* positive control (DE06 x LV01 cross with natural bacterial assemblage) and (b) with the addition of *Marinobacter* sp. DG879 (Gc/Mar).

Toxin profiles (Day 25) were generally very consistent among replicates (Fig. 4). Parent strain GCDE06 produced higher proportions of C1+2 (F = 63.21, df = 9, p = <0.001) and GC1+2 (F = 42.42, df = 9, p = <0.001) and lower proportions of C3+4 (F = 53.96, df = 9, p = <0.001), GTX2+3 (F = 6.59, df = 9, p = 0.033), and GTX1+4 (F = 5.41, df = 9, p = 0.048) than strain GCLV01 (Fig. 4). The toxin profile of the majority of offspring cultures were most similar to that of the GCDE06 parent cultures, with the exception of those grown with *Marinobacter* sp. DG879 which exhibited lower proportions of C1+2 (F = 435.35, df = 9, p = <0.001) and C3+4 (F = 38.7, df = 9, p = <0.001) and greater proportions of GC1+2 (F = 46.19, df = 9, p = <0.001) and dcGTX2+3 (F = 297.40, df = 9, p = <0.001) (Fig. 4). None of the cultures contained detectable concentrations of dcSTX, STX or GC3. Example HPLC chromatograms from cultures grown with a mixed bacterial community and with *Marinobacter* sp. DG879 are shown in Figure 5. Offspring positive control cultures were dominated by C2 with dcGTX3 as a minor component, whereas dcGTX3 was a major component and C2 a minor component in cultures grown with *Marinobacter* sp. DG879.

**Figure 6:**
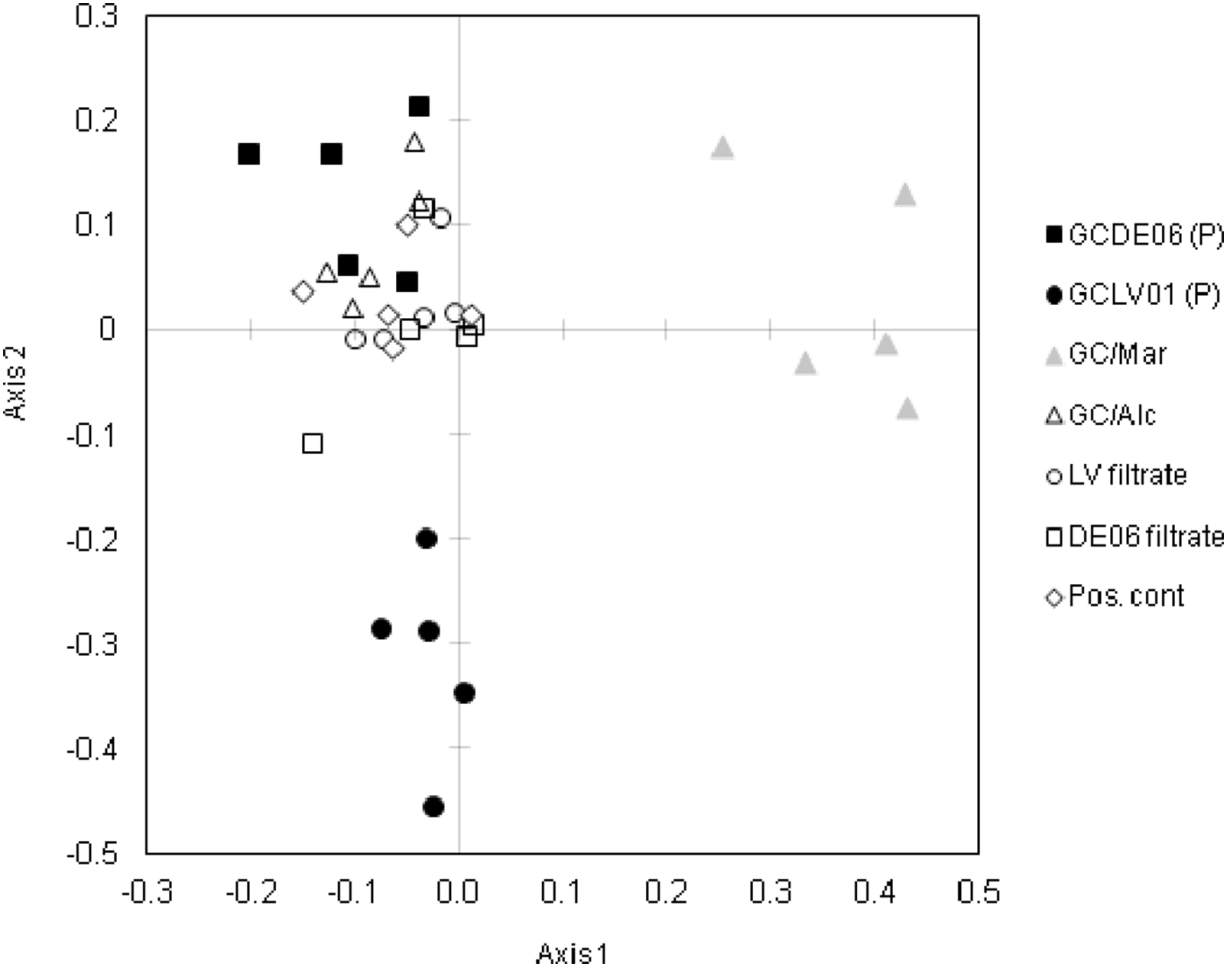
Canonical discriminant analysis of principal coordinates of toxin profiles of *G. catenatum* parent and offspring cultures.

The CAP analysis of toxin profile data for all 35 cultures resolved three distinct toxin profile groupings. Parent cultures GCDE06 and GCLV01 were clearly separated along axis 2 (y-axis), primarily due to the different ratios of C1+2 versus C3+4 (Fig. 6). Cultures grown with *Marinobacter* sp. DG879 clustered separately along axis 1 (x-axis), and all other offspring cultures clustered with or near parent GCDE06, or between the GCDE06 and GCLV01 parent clusters (Fig. 6).

## Discussion

### Bacterial effects on toxicity of *G. catenatum*

A number of previous studies have also shown that bacteria change or induce cellular toxicity of axenic and non-toxic algal strains [12], [18], [34], [35], [36] however our study is the first to show that altering the culture-associated bacterial community results in changes to both PST content and PST production rate. The toxin content of *G. catenatum* vegetative cells varies considerably [4], [21], [37] and is influenced by a range of culture-related and environmental factors such as isolation method, growth medium, nutrient type, concentration, salinity and temperature [21], [38], [39], [40]. All of these factors were controlled across our treatments, therefore our data shows that change in both cellular toxicity, net PST production and PST profile of the dinoflagellate is due to the modification of the bacterial community. Culture-based microbial diversity of the uni-bacterial cultures routinely recovered only the single bacterial type added in each case (*Alcanivorax* or *Marinobacter*) and we are confident that the cultivable communities were overwhelmingly dominated by the added bacterial strain.

Previous work shows that *G. catenatum* culture bacterial communities are typically composed of 15–24 distinct bacterial genotypes and a consistent community structure dominated by α- and γ-Proteobacteria and Bacteroidetes [11], [24]. While we do not have detailed microbial community data for the mixed community cultures in the present experiment, it is reasonable to assume similarly complex communities associated with parent and the mixed community offspring cultures. The observed changes in toxicity could then be related to changes in bacterial associate composition (i.e. the specific bacteria present), the reduction in community complexity, or both. Which factor is most important is difficult to determine from our experimental design and data, however it is interesting to note that the two uni-bacterial cultures showed the lowest cellular toxicity suggesting that reduced bacterial diversity may be partially responsible for reduced toxin content of *G. catenatum*. Axenic and antibiotic treated *A. catenella* and *A. tamarense* cultures with substantially reduced bacterial density and diversity also show reduced PST content [18], [41] indicating that this response is not confined to *G. catenatum.*

Earlier studies of *G. catenatum* have shown up to 40-fold reduction in the cellular toxicity of *G. catenatum* cultures established from wild resting cysts [21]. We did not observe the same scale of reduction in cyst germinated offspring, however, we did detect an 8-fold reduction in mean net toxin production rate (amol cell^−1^ day^−1^) compared to parent cultures. Studies of PST profiles of resting cysts indicate that PST production is reduced or stops during gamete and resting cyst formation [42], [43]. Sonication and washing of cysts during isolation may remove specific microbial associates (and their metabolites) required to fully induce dinoflagellate PST synthesis pathways after germination [21], [24]. Alternatively, reduced PST production may be associated with down-regulation of many secondary metabolite synthesis pathways during cyst formation and dormancy, or a form of reversible gene-silencing as described in some mycotoxin-producing fungi [44], [45].

The strong linear relationship between specific toxin production rate (*µ_tox_*) and specific growth rate (*µ*), and the poor correlation between net toxin production rate (*R_tox_)* and *µ* found for *G. catenatum* in this study, is similar to that of *Alexandrium fundyense* [30]. Anderson et al. [30] suggested this pattern resulted from nutritional deficiencies in batch cultures, causing an uncoupling of toxin synthesis from cell division, leading to highly variable rates of toxin accumulation. We sought to minimise nutritional limitation by harvesting cultures while in logarithmic growth and using saturating light intensity for *G. catenatum* at 21°C (80–90 µmol PAR m^−2 ^sec^−1^) [46], however, absolute growth rates observed in our experimental cultures are relatively low compared to other culture studies at similar temperatures and some of our cultures may have been light-limited leading to nutrient-limited growth in some cases. Alternatively, PST synthesis has been shown to occur only during the G1 phase of cell division [47], therefore the poor correlation may be due to differences in division synchrony among cultured treatments. Secondary metabolites (such as toxins) are also often subject to induction effects (up regulation) in response to unfavourable conditions [48], [49], further reducing the likelihood of a simple correlation with growth rate. Current knowledge of saxitoxin gene expression in dinoflagellates is limited, but recent studies suggest transcriptional or pre-translational regulations are major components. Taroncher-Oldenburg and Anderson [49] identified more than 20 transcriptionally regulated genes that were differentially expressed throughout the cell cycle of *Alexandrium fundyense* which were either up- or down regulated during toxigenesis. Similarly, Zhuang et al. [50] found that genes related to toxin production were expressed at different levels at different time points of the diel cycle.

### Bacterial effects on toxin profile

The dominance in our cultures of sulfocarbamoyl toxins C1+2 and C3+4 and the more recently characterised hydroxy-benzoate toxins (GC1+2) is consistent with the range of toxins evident from earlier studies of Australian *G. catenatum* [4], [21]. The substantially different profile produced by cultures grown with *Marinobacter* sp. DG879 (Gc/Mar; lower C1+2 and C3+4; greater dcGTX2+3 and GC1+2) in comparison to the other cultures, could be due to conversion of C1 and C2 to their decarbamoyl derivatives dcGTX2 and dcGTX3. The GC-toxins, GC1 and GC2 are *p*-hydroxybenzoate analogues of the carbamate toxins GTX2 and GTX3 (respectively) but the biosynthetic pathway of GC-toxins is not yet fully understood [9], [10], [23].

Whether the observed PST congener transformations are carried out by *G. catenatum* only in the presence of *Marinobacter* sp. DG879 or by the bacterium itself is not clear. Bacterial PST production has been speculated or suggested by many studies [51], [52], [53] but there is currently no convincing structural evidence to confirm PST production by heterotrophic bacteria. Our previous studies have also shown that *Marinobacter* sp. DG879 does not produce PST-like toxins or activity [24] therefore the PST profile changes observed in GC/Mar cultures is not explained by production of particular PST congeners by *Marinobacter*. The recent discovery of dinoflagellate homologues of saxitoxin synthesis genes (e.g. SxtA1, SxtA4, SxtG) in *Alexandrium*, *Pyrodinium bahamense* and *Gymnodinium catenatum* [9], [10], [54] also appears to confirm that saxitoxin synthesis in our cultures is primarily or exclusively of dinoflagellate origin.

The changes may be mediated by bacterial enzymatic transformation of PSTs [14], [15], [16]. For example an uncharacterised carbamoylase activity has been demonstrated in bivalve-associated gut enzymes transform GC1, GC2 and GC3 to the more toxic dcGCX2, dcGTX3 and dcSTX [16]. This pathway could explain transformation within *G. catenatum* of C1+2 to dcGTX2+3, but does not explain the high proportions of GC1+2. On the other hand, proportional increase in GC1 and GC2 is balanced by a similar reduction in C1 and C2, suggesting separate biosynthetic pathways or more complex enzymatic transformations. To date all bacterial PST transformation activities in earlier studies have, however, been observed in supernatants of bacterial cultures or shellfish extracts. This indicates that activity is mediated by extracellular enzymes, presumably only acting on PST in the surrounding medium or cell boundary layer. We examined only intracellular PST in this work therefore any bacterial mediated PST transformation would need to be mediated by enzymes produced by intra-cellular bacteria or by surface bacteria releasing enzymes that are actively transported into the dinoflagellate cell. Alternatively particular bacterial associates may produce metabolites that have an indirect influence of *Marinobacter* sp. DG879 via their action on toxin synthesis pathways or physiology of the dinoflagellate cell.

Multivariate comparison of parent PST profiles with the 25 offspring from our experiments contradict previous parent-offspring studies of *G. catenatum* [5] and *Alexandrium* species [55], [56] which all report segregation of parental toxin profiles in a 2∶2 Mendelian pattern. If this was the case for our study, the CAP ordination should show offspring clustering with or near the parent profiles in equal approximately numbers. Excluding the GC/Mar offspring that cluster separately on the axis 1 of the CAP, the majority of other offspring profiles cluster almost exclusively with parent GCDE06 (see Fig. 6) and indicate a uni-parental inheritance pattern in our experiment.

Resting cysts were not produced in self-crosses of either parent culture so it is highly unlikely that a majority of the offspring were products of self-crosses of GCDE06. A 2∶2 segregation pattern also assumes the major determinants of toxin profile are inherited as single locus. Given the complexity of the core saxitoxin biosynthetic pathway (>20 genes), the additional predicted tailoring enzymes, and also a probable presence of multiple expressed copies [10], it would in retrospect be surprising to find that PST profiles were inherited as a single non-recombining unit in dinoflagellates.

Our controlled bacterial community manipulation and germination study demonstrates that interactions with bacteria result in distinct and reproducible changes in cellular toxicity and toxin production rate, and cause significant changes to the intracellular PST profile of *G. catenatum*. How the observed changes are mediated remains unclear, however the balance of evidence suggests bacterial influences on dinoflagellate cell physiology and growth are the most likely mechanism.
